# Exploring personalized psychotherapy for depression: A system dynamics approach

**DOI:** 10.1371/journal.pone.0276441

**Published:** 2022-10-27

**Authors:** Andrea K. Wittenborn, Niyousha Hosseinichimeh

**Affiliations:** 1 Department of Human Development and Family Studies, Michigan State University, East Lansing, MI, United States of America; 2 Division of Psychiatry and Behavioral Medicine, Michigan State University, Grand Rapids, MI, United States of America; 3 Department of Industrial and Systems Engineering, Virginia Tech, Blacksburg, VA, United States of America; Istituto Superiore Di Sanita, ITALY

## Abstract

Depressive disorders are the leading contributor to medical disability, yet only 22% of depressed patients receive adequate treatment in a given year. Response to treatment varies widely among individuals with depression, and poor response to one treatment does not signal poor response to others. In fact, half of patients who do not recover from a first-line psychotherapy will recover from a second option. Attempts to personalize psychotherapy to patient characteristics have produced better outcomes than usual care, but research on personalized psychotherapy is still in its infancy. The present study explores a new method for personalizing psychotherapy for depression through simulation modeling. In this study, we developed a system dynamics simulation model of depression based on one of the major mechanisms of depression in the literature and investigated the trend of depressive symptoms under different conditions and treatments. Our simulation outputs show the importance of individualized services with appropriate timing, and reveal a new method for personalizing psychotherapy to heterogeneous individuals. Future research is needed to expand the model to include additional mechanisms of depression.

## Introduction

Depressive disorders are the leading contributor to medical disability [1], yet only 22% of depressed patients receive adequate treatment in a given year [2]. About 48% of people with depression don’t receive care and, of those who do, response to treatment varies widely, and poor response to one treatment does not signal poor response to others [3]. Only half of patients find relief with a first-line psychotherapy [4,5], yet half of patients who try a second option improve. Unfortunately, three-fourths of patients do not remain in treatment long enough to try an alternative approach when an initial treatment fails, illustrating the importance of identifying the optimal intervention approach early [6].

While attempts to personalize psychotherapy to patients have produced better outcomes than usual care [3,7,8], research on personalized psychotherapy is still in its infancy. In one approach, studies have used pre-treatment variables to match patients to different psychotherapies and patients with a better match reported better outcomes [7,9]. Another approach tested the effects of matching psychotherapy to patients’ strengths versus their weaknesses, and findings showed that personalizing treatment to patients’ strengths resulted in better depression outcomes than selecting treatment based on patients’ deficits [10]. Building on this work, DeRubeis and colleagues [8] developed the Personalized Advantage Index (PAI) to predict whether psychotherapy or antidepressant medication would produce a better outcome for a given patient based on five pre-randomization variables; analyses of existing data revealed the PAI’s potential for improving outcomes. Existing methods for personalizing care rely on several pre-treatment patient characteristics (e.g., age, comorbid personality disorder, intake symptomatology), and no known approaches to date have attempted to link a patient’s mechanisms of disease to the mechanisms of action of a treatment–a method that has been useful in personalizing pharmacotherapy [11].

Depression is a highly complex condition that involves a large number of interacting mechanisms, thereby requiring a method capable of representing multiple non-linear interactions. One method for understanding complex problems and the impact of potential interventions is system dynamics (SD) simulation method. SD is a method that has been used to identify ways to manage and prevent chronic diseases, mostly at the population-level, and for public health more generally [12,13]. SD models can represent the many non-linear interactions that contribute to a problem over possibly distinct time scales [14]. Through a process of conceptual, mathematical, computational, and simulation modeling, SD makes it possible to examine different aspects of systemic complexity and simulate what would happen to a person or population under various circumstances in a virtual environment. When using detailed datasets to ensure the simulation resembles its real-world counterpart, SD can be used to develop decision-support tools in which one can assess "what would happen if" an intervention or policy was implemented [15]. In contrast to traditional clinical trials, the use of a virtual environment in SD makes it possible to simultaneously examine many interventions (e.g., the effect of one treatment can be ‘erased’ from a virtual patient before testing another), clinical targets, moderators, and patient characteristics. SD is particularly relevant for depressive pathogenesis, where many variables have non-linear interactions, and some may operate at the scale of days while others operate over weeks, months, or years [16].

A central element to SD modeling is to identify and operationalize ’feedback loops’. Feedback loops are particularly important because they regulate dynamic phenomena [17] and can amplify small individual differences, thus contributing to the large heterogeneity of depression. One example of a feedback loop occurs when economic hardship increases vulnerability to depression, and depression contributes to loss of energy and motivation, which in turn leads to job loss and further exacerbates economic hardship. To identify the feedback loops contributing to depression, we used SD to develop a qualitative model incorporating the biological, cognitive, social, and environmental reinforcing mechanisms of depression, including key inertial factors thought to contribute to its development [16]. Building off this research and following recommendations for phased model building, a second study calibrated and validated an SD model of rumination, stress, and depression [18]. The SD model was used to examine the progression of depressive symptoms among heterogeneous adolescents. Findings showed that changes in the initial value of prior stressors and rumination as well as current stressors generated diverse trends in depressive symptoms and highlighted the importance of personalized intervention.

The present study aims to use the SD model of depression, rumination, and stress to explore a method for personalizing psychotherapy. Specifically, this study will examine whether mindfulness-based cognitive therapy (MBCT) has a different effect on adolescents who differ by prior life stressful events, ongoing stressors, rumination, and trajectories of depressive symptoms. Since people often delay seeking treatment for up to eight years after experiencing a first episode of depression [19], this study will also examine the effects of various delays in seeking treatment (i.e., 6 months, 2 years, 4 years, and 8 years after a first episode) on trajectories of symptoms among heterogeneous adolescents. Each simulation will compare the effects of therapy or therapy timing on 2,500 heterogeneous adolescents (i.e., 32 unique patient profiles). This is the first known study to use SD simulation modeling to explore personalized psychotherapy.

## Method

This study was declared exempt by the Michigan State University Institutional Review Board.

### Participants

Participants were 1,065 adolescents (*n* = 520 female; *n* = 545 male) from grades 6 to 8 in two middle schools in central Connecticut. The sample included 56.9% (*n* = 610) Hispanic/Latino, 13.2% (*n* = 141) non-Hispanic White, 11.8% (*n* = 126) non-Hispanic Black, 9.3% (*n* = 100) biracial/multiracial, 2.2% (*n* = 24) Asian/Pacific Islander, 0.8% (*n* = 9) Middle Eastern, 0.2% (*n* = 2) Native American, and 4.2% (*n* = 45) other racial/ethnic groups. Based on school records, 62.3% of students were eligible for free or reduced lunch. The socioeconomic status of the community in which the two schools are located is rated low because the income per capita is $18,404.

### Procedure

This study used existing data collected by McLaughlin and Nolen-Hoeksema [20]. A full description of the procedures are available in Michl et al. [21], however, a brief summary of data collection procedures is included below. The parents of eligible adolescents (*n* = 1,056) were contacted to provide active consent. Parents who did not return the consent form were then contacted by phone. About 22% of parents could not be reached by phone, and six parents refused to let their child participate in the study. Adolescent participants provided written assent. The participation rate at baseline was 72%. Two additional assessments were conducted after the first assessment. The first and second assessments were four months apart, and the second and third assessments were three months apart. Of those who completed the first assessment (T1), 28% (*n* = 221) did not complete the second assessment (T2), and 20.4% (*n* = 217) did not complete the third assessment (T3). The primary reason for attrition was due to students leaving the school district. Those who did not complete the second and third assessments were more likely to be female (*χ*^2^ (1) = 6.85, *p* < 0.01), however, they were not different in terms of grade level, race/ethnicity, or being from a single-parent household (*ps* > 0.10). Depressive symptoms and rumination level of those who did not complete at least one of the follow-up assessments did not differ from those who completed all assessments (*ps* > 0.10). Depressive symptoms and stressful life events were collected at T1 and T3 while the level of rumination was measured at all assessment times (T1, T2, and T3).

### Measures

#### Stressful life events

The Life Events Scale for Children [22] includes 25 instances of stressful life events. Participants are asked to indicate if they experienced any of the events in the past six months (e.g. “Your parents got divorced” and “You got suspended from school”). This measure has a high test-retest reliability over a two-week period [23,24].

#### Rumination

The Children’s Response Style Questionnaire (CRSQ) [25] is composed of 25 items that measure the level of rumination, distraction, and problem solving in response to sad feelings. The CRSQ are grouped into three scales: 1) ruminative response subscale, 2) distracting response subscale, and 3) problem-solving subscale. We used the ruminative response subscale (CRSQ-Rumination) which contains 13 items and generates a score between 13 and 52. Adolescents are asked to indicate how often they respond in a given way when they feel sad (i.e., *almost never* = 1, *sometimes* = 2, *often* = 3, or *almost always* = 4). Sample items include “Think about how alone you feel,” “Think about a recent situation wishing it had gone better,” and “Think why can’t I handle things better?” The CRSQ-Rumination exhibited good reliability in this sample (*α* = 0.86) and in previous research [25].

#### Depressive symptoms

The Children’s Depression Inventory (CDI) is a 27 item measure of depressive symptoms in children and adolescents [26]. Each item contains three statements from which respondents choose the one that best describes them in the past two weeks (e.g., “I am sad once in a while,” “I am sad many times,” and “I am sad all the time”). The human subjects committee and school officials requested that the item related to suicide be removed from the questionnaires. The remaining 26 items generate a score between 0 and 52. The CDI exhibited good reliability in this sample (*α* = 0.82).

### Analysis: Developing a system dynamics model of depression

Our SD simulation model of depression simultaneously captured the reciprocal relationships among depressive symptoms, rumination, and stressors at the individual level. SD models are often used to understand complex systems and the endogenous feedback mechanisms underlying observed trends [14]. This method has provided remarkable contributions in various disciplines, including health policy and research [27–33], and it is very useful for capturing the feedback mechanisms underlying depression [16]. SD models include a set of ordinary differential equations that can be simulated to examine the results of different assumed model structures. The strength of the hypothesized causal pathways can be determined by statistical estimation of these models. Exogenous random terms can be included in the formulation of SD models.

Using SD models to study depression provides three advantages. First, SD models can capture multiple feedback mechanisms, latent variables, and accumulations. Although simultaneous equation methods and structural equation models can include feedback and latent variables, SD models incorporate all of these and also allow for nonlinearities. Second, SD models provide insights about the behavior of the system by showing the simulation results over continuous time. Third, SD models incorporate broad and realistic feedback mechanisms that allow for designing and analyzing different interventions for the same simulated individual.

**Fig 1:**
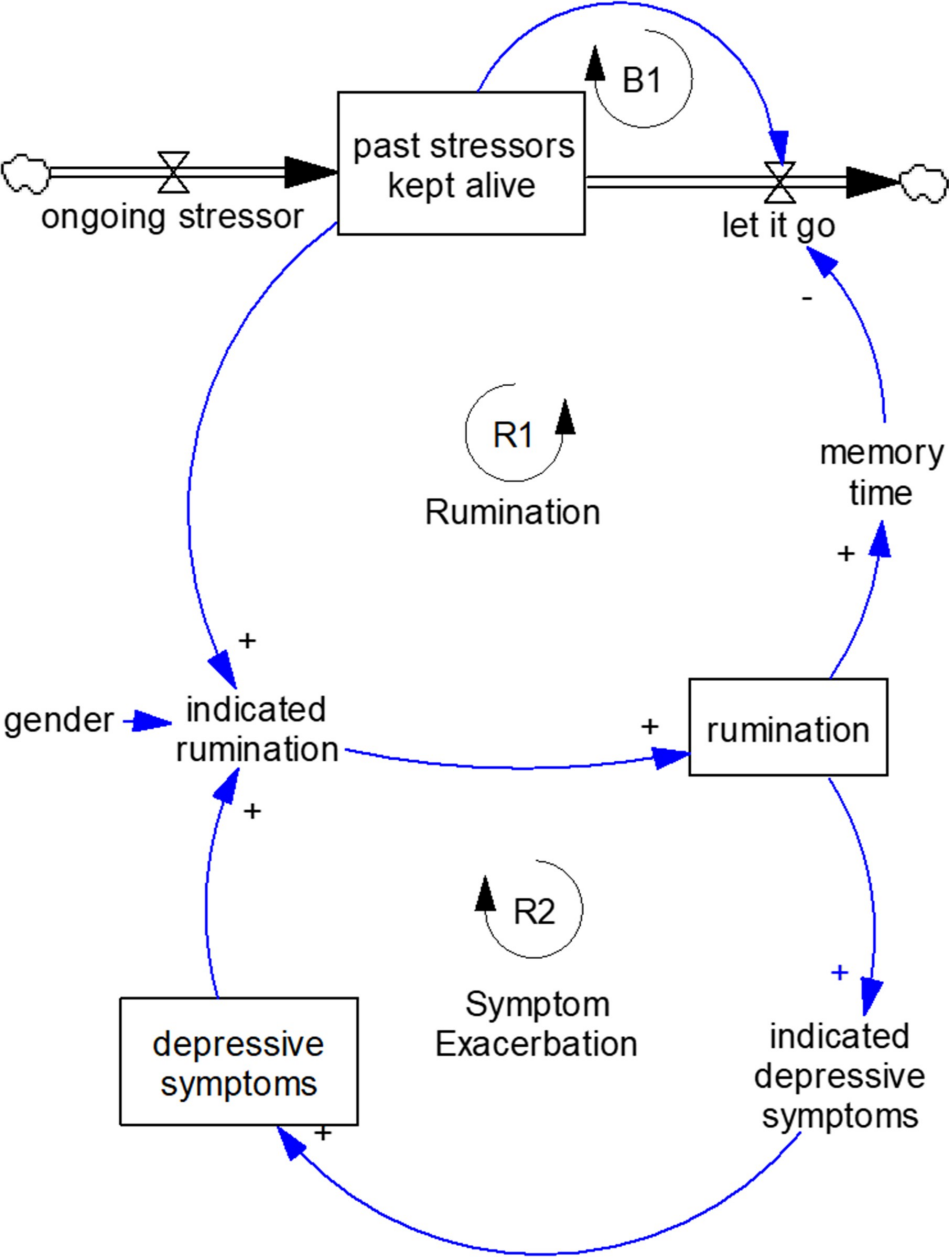
The depression-rumination conceptual model. Boxes depict stock (or state) variables and arrows with valves represent flows into/out of the stocks. Single-line arrows indicate causal relationships hypothesized among variables (the strength of which is estimated below). A stock variable is the accumulation of the difference between its inflows and outflows and, mathematically, is represented as an integral. The outflow, let it go, subtracts from the stock and represents a negative causal connection, which is not shown on the diagram.

The SD model that we developed in this study included two reinforcing feedback loops (Fig 1). It is based on the response style theory [34] depicted in reinforcing feedback loop 2 (R2), and the mechanism suggested by Ruscio et al. [35] shown in R1. The assumption behind R1 is that after experiencing a stressor, a person with a ruminative style spends time ruminating about it which keeps those stressors active, and thus leads to an even higher level of rumination. In other words, rumination increases vulnerability to stressors by keeping an individual activated and the stressor “alive” [35]. R2 captures the finding in previous research that a higher level of *rumination* causes more *depressive symptoms* and more *depressive symptoms* leads to even more *rumination* [34]. The balancing loop B1 shows the process in which people let the stressor go. As the stock *past stressor kept alive* increases, the outflow *let it go* elevates which leads to lower past stressors kept alive.

The formula of the model is as follows. *Past stressors kept alive* and *depressive symptoms* influence *rumination* with a delay. Thus, current *rumination* is the first order delay (weighted average) of *indicated rumination*. *Indicated rumination* is a linear function of *past stressors kept alive* [21], *depressive symptoms* [34], and *gender* [36].


$$indicated\;rumination_{t}=(\theta_{1}+\theta_{2}\times depressive\;symptoms_{t}+\theta_{3}\times gender+\theta_{4}\times past\;stressor\;kept\;alive_{t})/(1-\theta_{5})$$ (1)

*Rumination* does not influence *depressive symptoms* immediately, as a result, *depressive symptoms* is formulated as the first order delay of *indicated depressive symptoms* that we assume is a function of *rumination*.


$$indicated\;depressive\;symptoms_{t}=(\theta_{6}+\theta_{7}\times rumination_{t})/(1-\theta_{8})$$ (2)

In addition, *indicated rumination* and *indicated depressive symptoms* are not deterministic and are influenced by random events outside the model boundaries such as unmeasured contextual influences. Usually, those events are serially correlated, as a result, we added first-order auto-correlated noise terms that are normally distributed to the *indicated rumination* and *indicated depressive symptoms*. In turn, *θ*_10_ and *θ*_11_ are the standard deviation of these noise terms and *θ*_12_ is the correlation time.

The stock of *past stressors kept alive* contains the memories of stressors not the stressors themselves (stock variables are defined in the legend in Fig 1). A stressful event occurs and ends at some point in time, but ruminating about it as well as its impact on depressive symptoms may last much longer. The variable constructed directly from the measure of stressful life events cannot be a good approximation for the *past stressors kept alive* because it reports stressful events that happened in the past six months. Participants may still ruminate about an event that happened more than six months ago or they may not think about an event that occurred less than six months ago. To overcome this limitation, *past stressors kept alive* and *ongoing stressors* were estimated from stressful life events (see procedure explanation in S1 Table). The stock of *past stressors kept alive* is the accumulation of the difference between its inflow (i.e., *ongoing stressors*) and outflow (i.e., the process through which people let the stressor go).


$$past\;stressors\;kept\;alive_{t}=\int_{t}[ongoing\;stressors(s)-let\;it\;go(s)]ds+past\;stressors\;kept\;alive_{t0}$$ (3)

$$let\;it\;go_{t}=\frac{past\;stressor\;kept\;alive_{t}}{memory\;time_{t}}$$ (4)

$$memory\;time_{t}=\theta_{9}\times rumination_{t}$$ (5)

All formulas are listed in S1 Table. It is important to note that the causal mechanism and the parameters of the SD model (θ_1_-θ_12_ in Fig 1) are the same for all individuals while the value of different stocks (e.g., depressive symptoms) and the random noises vary across individuals. The parameters of the model (See Table 1) were estimated by using the indirect inference method. The estimation procedures of this model are explained in detail in Hosseinichimeh et al. [37].

**Table 1:** Estimated parameters using indirect inference.

Unknown Parameters	Estimated parameters
Rumination Constant (θ_1_)	-1.2504 (0.991)
Effect of depression on rumination (θ_2_)	0.4236 (0.301)
Gender Coefficient (θ_3_)	2.5152 (1.002)*
Effect of stress on rumination (θ_4_)	0.2518 (0.117)*
Rumination Coefficient (θ_5_)	0.1639 (0.495)
Depression Constant (θ_6_)	0.3730 (0.039)*
Effect of rumination on depression (θ_7_)	0.0699 (0.003)*
Depression Coefficient (θ_8_)	0.8894 (0.004)*
Effect of rumination on time constant (θ_9_)	1.4741 (0.051)*
RumNoise Standard Deviation (θ_10_ = $\sigma_{r}^{2}$)	7.8735 (4.088)
DepNoise Standard Deviation (θ_11_ = $\sigma_{d}^{2}$)	0.0002 (0.016)
Correlation Time (θ_12_)	1.6008 (0.793)*

Standard errors are presented in parentheses.

*significant at 95% level.

## Results

Means and standard deviations of all measures at each evaluation time for all participants (*n* = 661) as well as separately for boys (*n* = 308) and girls (*n* = 353) are listed in Table 2. Girls reported more depressive symptoms at Time 1 (*p* = 0.03) and Time 3 (*p* = 0.08) and higher levels of rumination at all evaluation times (*p* = 0.00). There is no gender difference in stressful life events at Time 1 (*p* = 0.98) and Time 3 (p = 0.27).

**Table 2:** Summary of measures.

Variable	Total	Girls	Boys	Gender difference
Depressive symptoms at Time 1	9.48 (6.28)	9.98 (6.45)	8.91 (6.04)	1.07**
Depressive symptoms at Time 3	9.78 (7.64)	10.28 (7.58)	9.22 (7.68	1.06*
Rumination at Time 1	11.59 (7.52)	12.78 (7.71)	10.23 (7.06)	2.55**
Rumination at Time 2	10.85 (7.62)	12.24 (7.98)	9.25 (6.86)	2.99**
Rumination at Time 3	9.95 (7.95)	11.49 (8.24)	8.19 (7.22)	3.29**
Stressful life events at Time 1	4.96 (3.32)	4.97 (3.14)	4.96 (3.52)	0.01
Stressful life events at Time 3	4.20 (3.70)	4.35 (3.48)	4.03 (3.93)	0.32

Standard deviations are in parentheses.

*significant at the 0.1 level.

**significant at the 0.05 level.

### Trajectories of depressive symptoms by characteristics of participants

Although the parameters of the model (θ_1_-θ_12_) are the same for all individuals, the model generates different trajectories for participants because the initial values of depressive symptoms, rumination, gender, prior stressors (i.e., initial value of *past stressors kept alive*), and ongoing stressors (i.e., inflow of *past stressors kept alive*) differ across individuals. A simulated individual with sizable ‘prior stressors’ has experienced many stressors in the six months prior to the beginning of the simulation and a simulated person with a large value of ‘ongoing stressors’ represents someone who experiences stressors during the entire simulation.

#### Trajectories for female participants

We ran a full-factorial simulation experiment to examine how depressive symptoms of diverse individuals evolve over 120 months. We used a timeframe of 120 months since almost all first depressive episodes remit within 120 months [38]. We changed four factors (i.e., initial rumination, depressive symptoms, prior stressors, and ongoing stressors) for girls and boys separately. As a result, sixteen female and sixteen male groups emerged. S1 Fig depicts the sixteen female groups and S2 Fig depicts the sixteen male groups, which represent heterogeneous clinical presentations as is common in the real world. We used the same inputs to run fully controlled simulations for each group. The high and low levels of rumination and depression were found by adding or subtracting one standard deviation to or from the mean. We added two standard deviations to the mean of prior and ongoing stressors to find the high levels and we subtracted one standard deviation from the mean of prior stressors to determine its low value. The low value of ongoing stressors was set to zero because the subtraction of one standard deviation from its mean was negative.

**Fig 2:**
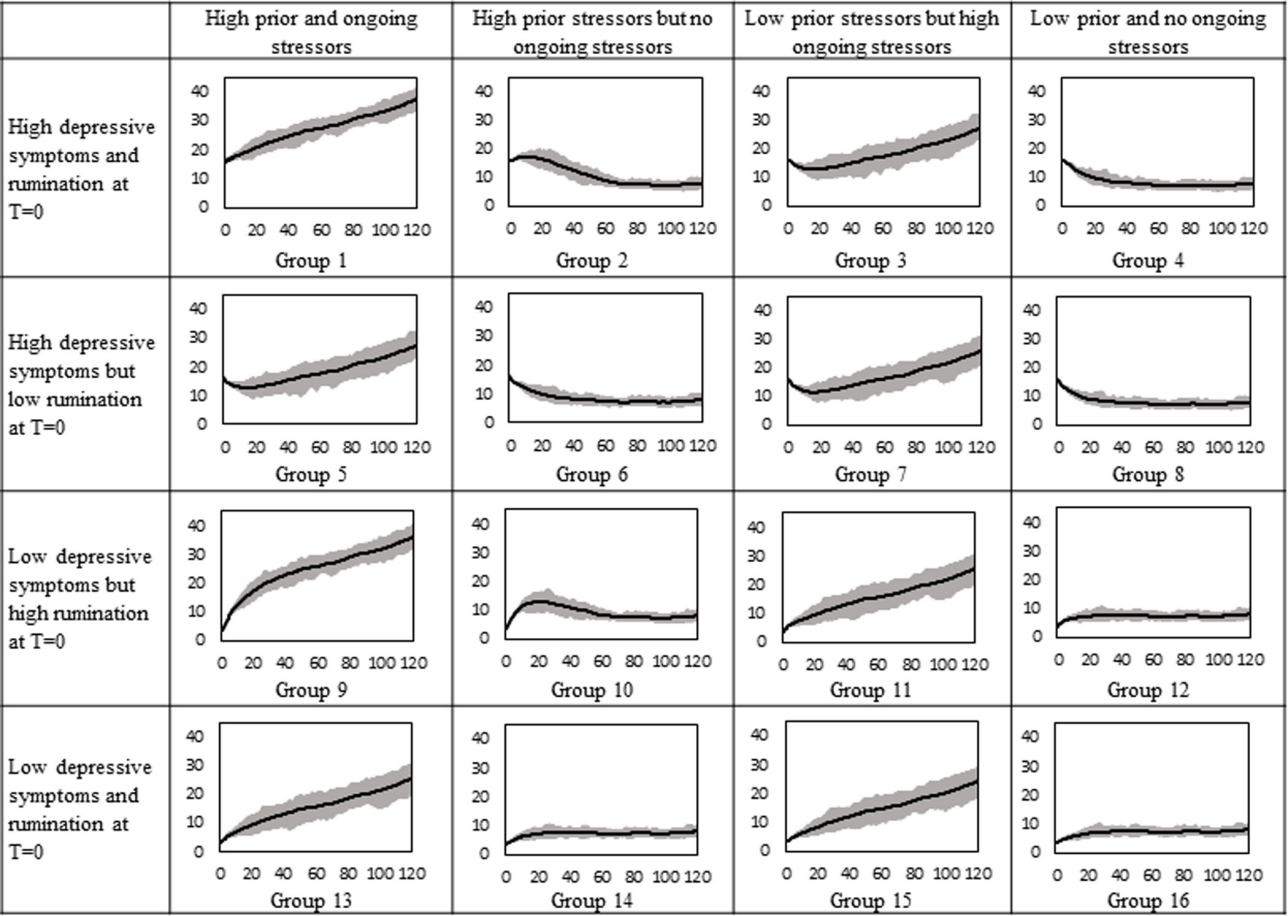
Simulated depressive symptoms over 120 months for 16 female groups.

**Fig 3:**
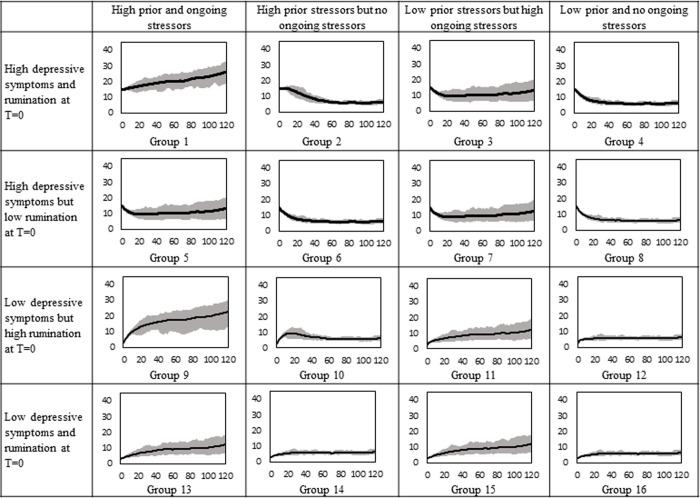
Simulated depressive symptoms over 120 months for 16 male groups.

As shown in Fig 2, the initial depressive symptoms of the first eight groups are high and values for the remaining groups are low. Individuals in groups 1 to 4 and 9 to 12 have sizable initial rumination while others have low rumination at the beginning of the simulation. For instance, individuals in group 1 have high initial rumination and depressive symptoms and they experienced stressful events in the past and additional stressors are occurring in their present life. In each group, we ran the model for 2,500 subjects with different random shocks to capture different environmental events that individuals encounter in the real world. At each point in time, we found the mean depressive symptoms of 2,500 subjects and the range enveloping 75% of the symptoms (Fig 2). The 75% envelope depicts the range including depressive symptoms between the 12.5 and 87.5 percentile within each group. The same procedures were followed for male adolescents (Fig 3). Based on Timbremont et al. [39], depressive symptoms above 16 is considered clinical depression for adolescents.

The major impact of ongoing stressors on depressive symptoms can be observed by comparing column 1 with 2 and column 3 with 4 in Fig 2. Depressive symptoms of girls with a high level of ongoing stressors (columns 1 and 3) increase over time while they follow a declining or stable trend for those with low ongoing stressors. For example, simulated adolescents in groups 1 and 2 are similar in terms of prior stressors and initial rumination and depression while they have different ongoing stressors. The depressive symptoms of group 1 exacerbate over time, which aligns with prior findings that indicate 6% of people who experience a depressive episode may not recover for 15 or more years [38]. In contrast, the symptoms of group 2 increase and then decline over 120 months. The initial increase of depressive symptoms for group 2 are caused by prior stressors that still exist in the stock of the *past stressors kept alive*. After a few months, rumination and depressive symptoms decrease because those stressors leave the stock (balancing loop B1 dominates the system) and no more stressors occur in the lives of participants in group 2. Depressive symptoms of group 2 reach a state of equilibrium after around 10 years because it takes a long time for group 2 to fully let go of the stressors. Girls in group 1, unlike group 2, experience stressors during the entire simulation. As a result, stressors accumulate in the stock of *past stressors kept alive* and rumination and depression reinforce each other and increase over time (both reinforcing loops R1 and R2 dominate the dynamics of the system). Prior stressors in combination with initial rumination influence the trajectory of depressive symptoms in the first few months. Depressive symptoms initially grow if both prior stressors and initial rumination are high because high initial rumination reduces *let it go* rates, thus, prior stressors stay longer in the stock of *past stressors kept alive* and rumination and depression intensify each other (R1 and R2 dominate). However, their impact is temporary and if there are no ongoing stressors, depressive symptoms decline and become stable over time (B1 dominates). For instance, group 10, which is high in depressive symptoms and ongoing stressors, experiences an initial increase in depressive symptoms while the depressive symptoms of group 12 or 14 only increase slightly to reach the steady-state level in the absence of any ongoing stressors. However, the interaction of initial rumination and prior stressors has a long-term impact if large levels of the two factors are accompanied by large ongoing stressors (groups 1 and 9). Group 9’s depressive symptoms are initially low but elevated initial rumination combined with large prior and ongoing stressors increase their symptoms to a level similar to Group 1 (Reinforcing loops R1 and R2 dominate the system).

#### Trajectories for male participants

The average and 75% envelope of the simulated depressive symptoms for 16 male adolescent groups are shown in Fig 3. The high/low levels of depression, rumination, prior stressors, and ongoing stressors for boys (15/3, 17/3, 12/1.4, and 2/0) are found by following the same procedures explained for girls. The high level of ongoing stressors was set at two for both genders to make the final trajectories of depressive symptoms comparable. The same level of ongoing stressors caused less depressive symptoms in boys than girls because girls have a higher tendency to ruminate about stressors.

### The impact of MBCT therapy on depressive symptoms

Next, we will describe results from our test of the effects of MBCT on the depressive symptoms of heterogeneous adolescents in a simulated environment. We developed a test based on the empirical literature in which a stressful life event with an intensity of 40 was applied at month 20 and continued for two months; MBCT was then received at month 30. Prior findings have shown that the impact of therapy may last up to 24 months [40]. Therefore, we hypothesized that MBCT reduced the ‘memory time’ by 60% from month 30 to month 54 [41]. Similar to the previous section, we simulated 2,500 individuals in each group and found the average and 75% envelope of the simulated depressive symptoms.

**Fig 4:**
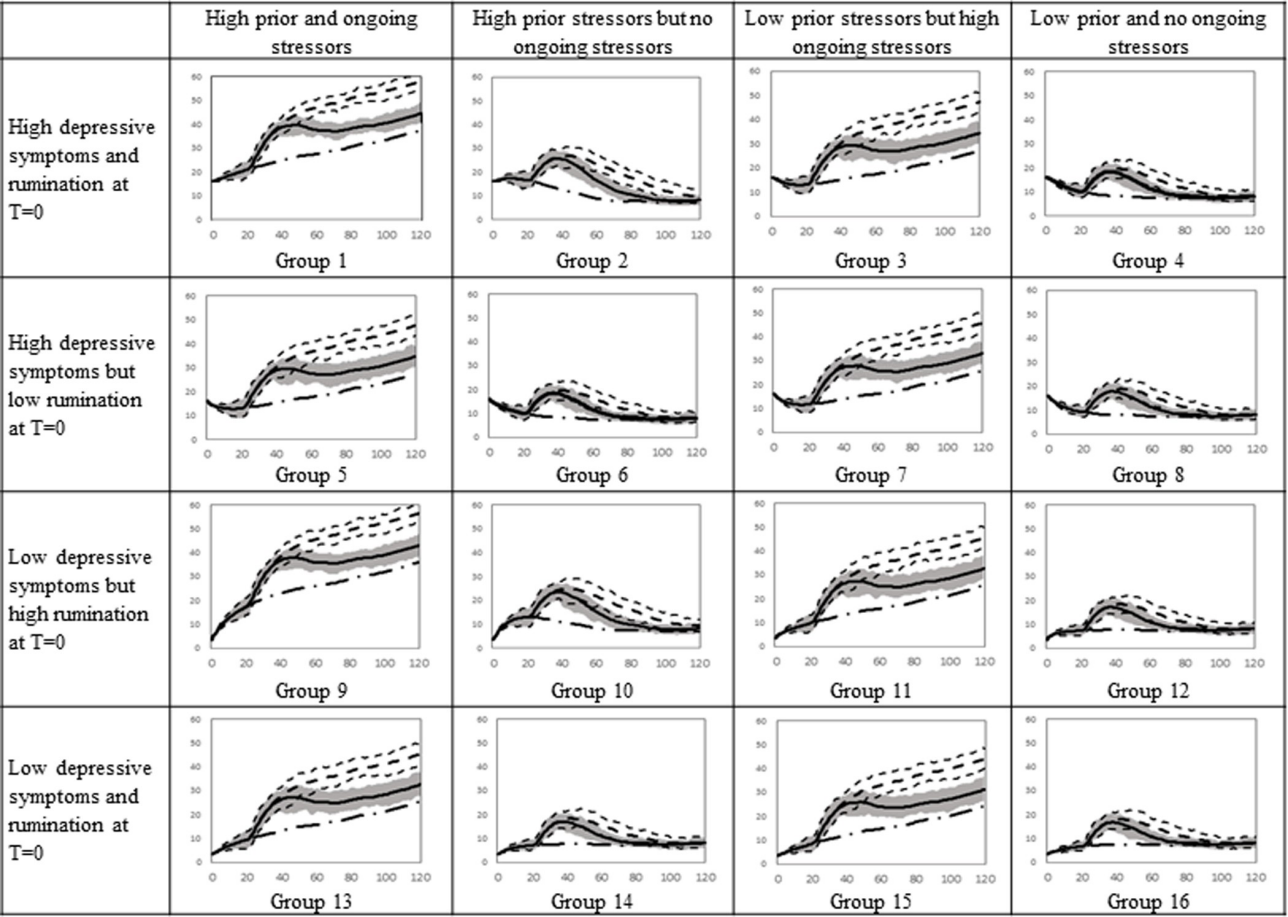
Simulated depressive symptoms for 16 female groups who experienced a stressful life event at month 20 and therapy at month 30. The long-dash-dot line captures the baseline output. The dashed line depicts the depressive symptoms when girls experience a stressful life event at month 20. The solid line shows the results when they experience a stressful life event at month 20 and then therapy at month 30.

The dashed line in Fig 4 shows the mean depressive symptoms when adolescents experience a stressful event beginning at month 20 without receiving MBCT and the dashed lines around it show the 75% envelope. The solid line depicts the symptoms when both the stressful event is added to the simulation at month 20 and MBCT begins at month 30; the 75% envelope is the gray area shown around it. For comparison, the baseline output from Fig 2 is included as a long-dash-dot line.

Comparison of columns 1 and 3 with 2 and 4 of the simulation results shows that those with ongoing stressors benefited the most from therapy. For instance, individuals in group 1 treated with MBCT after the intense stressful event (solid line in Fig 4) have much lower symptoms than those who did not receive the treatment after the stressor (dashed line in Fig 4). For group 2, although the difference between the symptoms with and without treatment is significant for a period of time, the symptoms among those receiving treatment and not receiving treatment merge near the end of simulation. The difference is larger for group 1 because stressors continue to occur in their lives and accumulate in the stock of ‘past stressors kept alive’. As a result, at any point in time, group 1 has a larger stock of past stressors and subsequently higher rumination and memory time to be treated with therapy. However, individuals in group 2 do not experience any stressors after month 20 and their stock of past stressors, thus, rumination and depression decline over time even without receiving therapy. Therefore, although group 2 experiences clinically significant declines in symptoms after therapy, MBCT has a higher impact for group 1. As the effect of therapy diminishes, the decline in symptoms slows and begins to increase around month 70.

**Fig 5:**
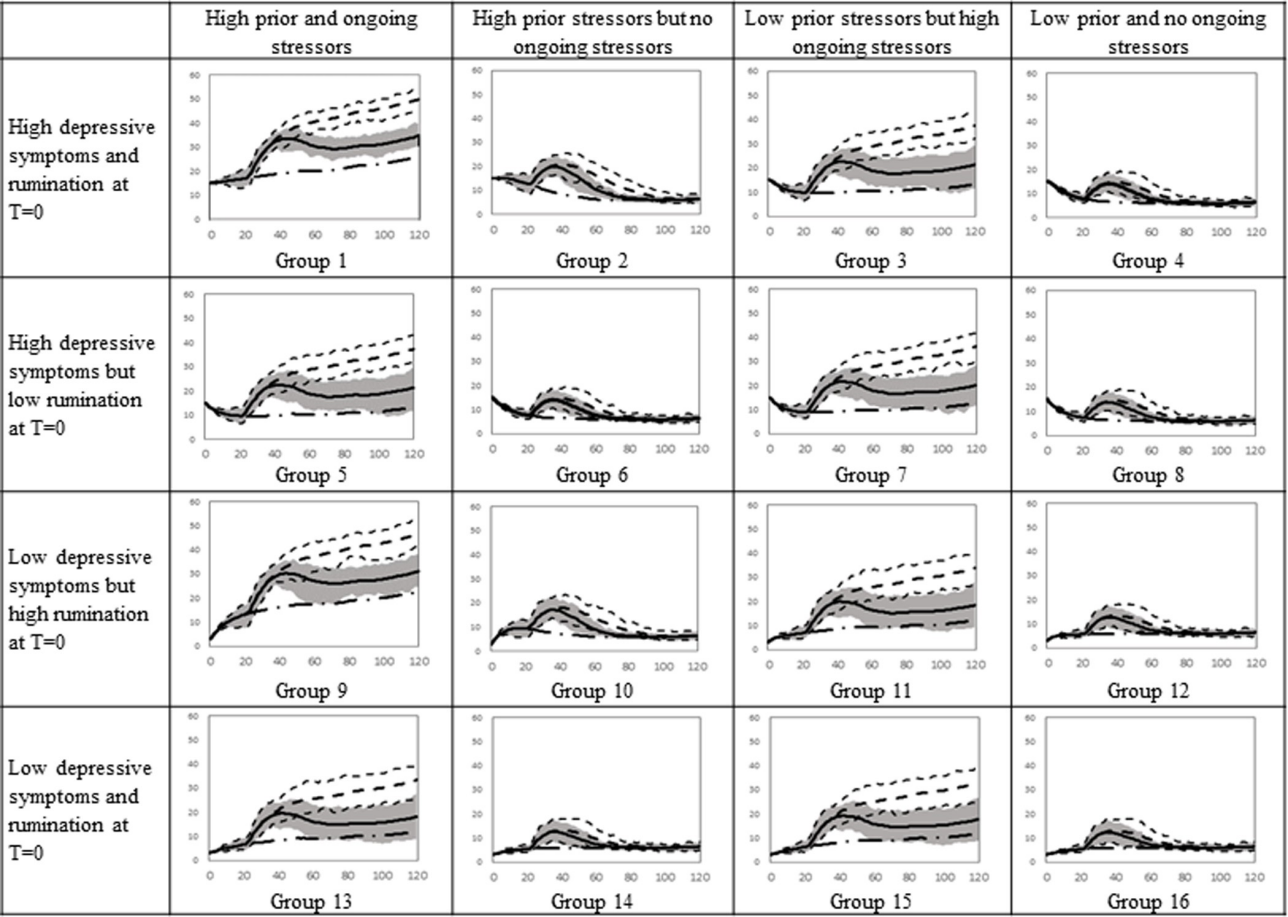
Simulated depressive symptoms for 16 male groups who experienced a stressful life event at month 20 and therapy at month 30. The long-dash-dot captures the baseline output. The dashed line depicts the depressive symptoms when boys experience a stressful life event at month 20. The solid line shows the results when they experience a stressful life event at month 20 and then therapy at month 30.

Similar to girls, boys with ongoing stressors received the most benefit from therapy (See Fig 5). The same level of ongoing stressors causes less depressive symptoms in boys than girls, as a result, the therapy has a higher impact on female adolescents. The overall trends of symptoms are the same for both genders because the underlying mechanisms are the same.

### Impact of MBCT based on the timing of initiation of treatment following an episode

**Fig 6:**
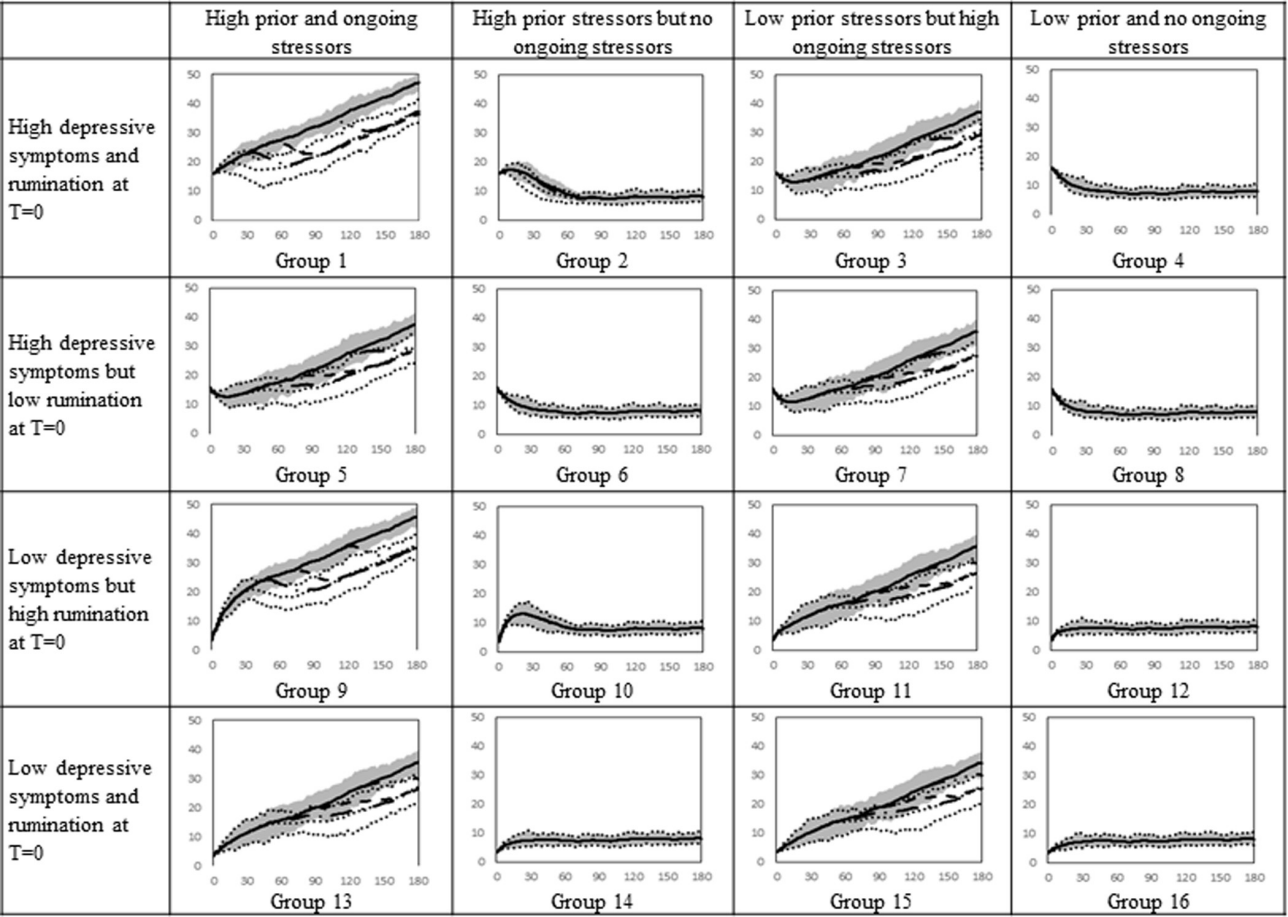
Simulated depressive symptoms for 16 female groups who receive therapy 6 months, 2 years, 4 years, and 8 years after their first episode. Solid line captures the baseline, the dot line, long-dash-dot, dashed line, and long-dash-dot-dot depict the depressive symptoms when girls receive therapy 6 months, 2 years, 4 years, and 8 years after their first episode.

**Fig 7:**
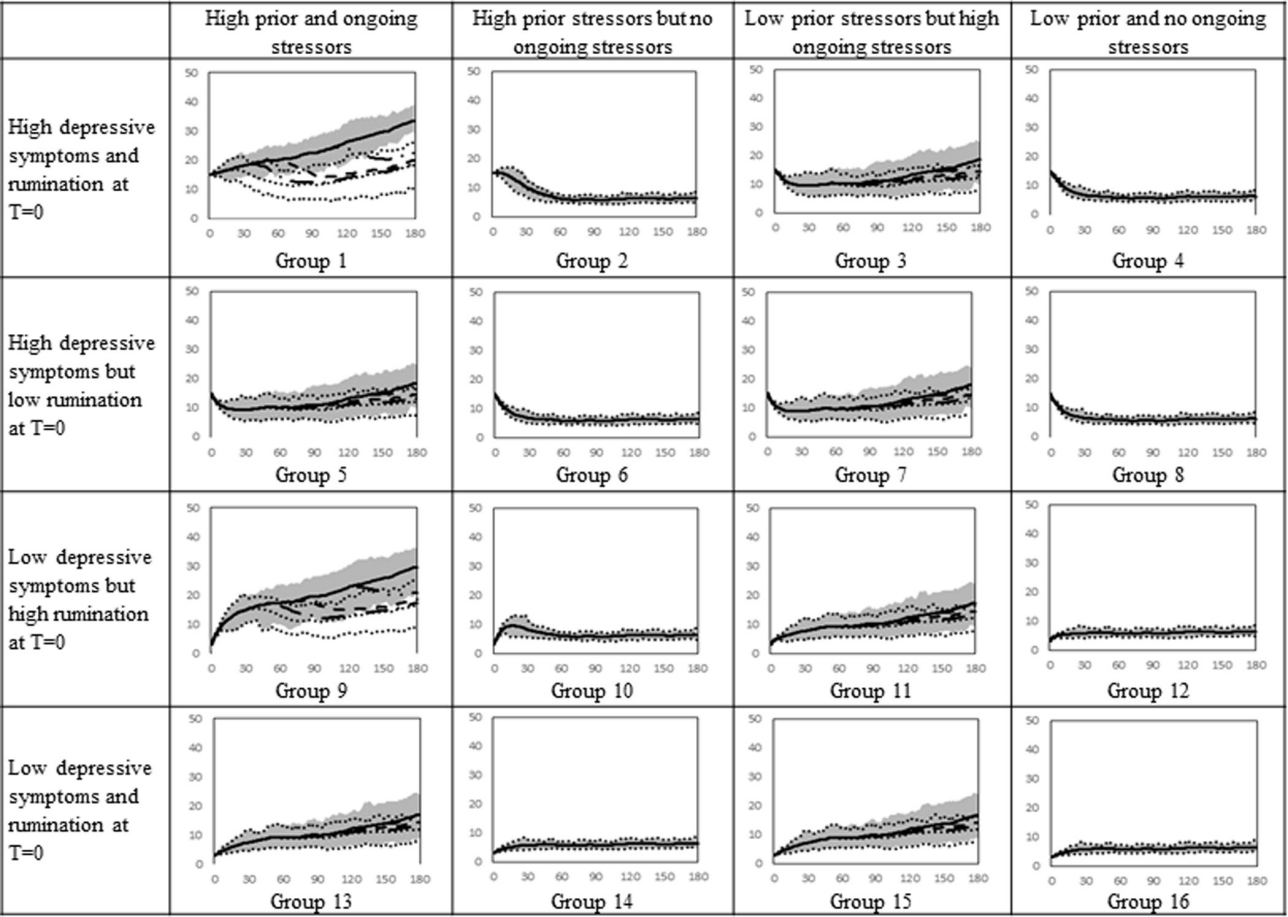
Simulated depressive symptoms for 16 male groups who receive therapy 6 months, 2 years, 4 years, and 8 years after their first episode. Solid line captures the baseline, the dot line, long-dash-dot, dashed line, and long-dash-dot-dot depict the depressive symptoms when boys receive therapy 6 months, 2, 4, and 8 years after their first episode.

Next, we investigated the impact of the timing in which MBCT was received. Specifically, we examined the effect of receiving MBCT beginning six months, two years, four years, and eight years after the first episode of depression, which is shown respectively with a dot line, dashed-dot line, dashed line, and dashed-dot-dot line in Fig 6 for females and Fig 7 for males. These time points were selected because people, on average, receive treatment 8 years after their initial episode [19], thus, this allowed us to examine the effects of receiving treatment then or sooner. To observe the impact of initiating therapy at different times following the first episode, the time horizon in the simulation was extended to 180 months. A depressive episode is defined as the persistence of depressive symptoms above 16 for at least two weeks. Again, we assumed that the therapy would reduce the ‘memory time’ by 60% and the effect of treatment would become noticeable after 2 months and last for 24 months. We simulated the model for 2,500 adolescents and reported the mean. We only showed the 75% envelope for the baseline output (receiving no therapy is shown with the gray shaded area) and for the group receiving therapy six months after the first episode (area is shown by dot lines) because the graphs become difficult to decipher.

Receiving therapy at any time after the first episode reduces depressive symptoms for girls with high ongoing stressors (See columns 1 and 3 of Fig 6, comparing the solid line with the other lines). However, if groups 1 and 9 who have elevated rumination and high prior and ongoing stressors receive therapy earlier, they would have significantly fewer depressive symptoms following the episode (for instance, compare the dot line with the dashed line in group 1 of Fig 6). Although the outputs of different therapy simulations eventually converge, the difference in symptoms after the therapy are clinically significant.

The trajectory of depressive symptoms for some adolescents who received therapy overlaps with the baseline for some individuals because they did not experience a depressive episode during the entire simulation (e.g., group 6 in Fig 6). Only boys with high prior stressors, ongoing stressors, and rumination received significant benefit from therapy (i.e., group 1 and 9 in Fig 7).

## Discussion

This study used an SD simulation model of depression developed and calibrated in Hosseinichimeh et al. [37] to investigate the effect of MBCT on depressive symptoms among heterogeneous adolescents. Simulation outputs showed that the level of ongoing stressors play a critical role in the evolution of depression and the interaction of the initial level of rumination and prior stressors influences the trend of depressive symptoms in the early months of the simulation. In one test, we applied an intense stressful event at month 20 followed by MBCT at month 30. We found that adolescents with high ongoing stressors benefited the most from therapy. Our model also demonstrated that the same level of ongoing stressors caused more depressive symptoms in girls. This is because girls are more likely to ruminate after experiencing a stressful event and the treatment has a higher impact on girls. However, it’s important to note that when comparing girls and boys, the overall trends of symptoms are the same because the underlying mechanisms for each gender are the same in the model. Therefore, regardless of gender, adolescents with ongoing stressors received the most benefit from therapy. We also examined the impact of the timing of treatment on depressive symptoms. Treatment at any point in time after the first episode reduced depressive symptoms for girls with high ongoing stressors but those who received therapy earlier after their first episode had a better quality of life. Therapy significantly reduced the depressive symptoms of boys if they had elevated levels of initial rumination and stressors. Our results are consistent with prior research findings that girls have a tendency to ruminate more than boys [36], and that interventions for rumination may be more beneficial for girls [42].

Our study makes several important contributions to the literature. Building on the literature in personalized mental health services [7,8], this study demonstrates preliminary support for a new method of examining personalized psychotherapy based on an individual’s active mechanisms of disease. Findings show it is feasible for SD simulations to explore various treatment paths among heterogeneous patients to assist in the selection of the optimal psychotherapy. Our model provides a simulation lab for testing the impact of treatment and timing of treatment on depressive symptoms of heterogeneous individuals. Future research that carefully builds on this model is needed to develop an SD model with additional mechanisms of disease to provide a more realistic lab for comparing treatment protocols. That said, our model is unique in that it captures the reciprocal relationships among stressors, rumination, and depressive symptoms simultaneously while previous research has only examined bidirectional relations between either rumination and depression [34] or stress and depression [43,44].

The current study highlights the value of personalized psychotherapy, including both prevention and treatment. Changes in prior stressors, ongoing stressors, and rumination have significant effects on the evolution of depressive symptoms. Our simulation indicates that depressive symptoms may aggravate rapidly among individuals with ruminative styles who face high levels of stressors, and such adolescents may benefit the most from timely treatment. However, the benefits of therapy diminish and the depressive symptoms of those with high ongoing stressors increases over time. For these individuals, booster sessions might be helpful in reducing the chance of recurrence. In addition, the same level of ongoing stressors generates more depressive symptoms in girls, as a result, gender should be considered as one of the critical inputs in tailoring treatments. Also, we showed that the depressive symptoms of an initially non-depressed individual exacerbates quickly if the adolescent has elevated initial rumination and high prior and ongoing stressors. This indicates the importance of screening and providing preventive care to non-depressed adolescents with ruminative styles who are facing stressors.

### Limitations

These findings should be interpreted in light of multiple limitations. First, depression is derived by multiple factors interacting in a complex feedback system [16]. Different cognitive, biological, genetic, social, and environmental mechanisms influence the evolution of depression. The present study only included stressors and rumination. To increase validity, we concentrated on adolescents to attempt to rule out the presence of some biological mechanisms that occur among adults with subsequent depressive episodes. Second, we used hypothetical numbers derived from the literature, including calculating one or two standard deviations from the mean of the variables in the model in order to investigate the impact of MBCT and the timing in which therapy was received. Ongoing stressors is a critical determinant of trajectories of depressive symptoms and the impact of therapy is sensitive to this variable. In one of the simulation experiments, we showed that only boys in two groups (i.e., group 1 and 9) benefited from therapy. It is possible this result could change if different levels of ongoing stressors were used. As such, we attempted to situate all decisions in the literature to increase validity and create the most realistic simulation lab possible.

### Conclusion

In this study, we developed an SD model of depression based on one of the major mechanisms of depression in the literature and investigated the trend of depressive symptoms under different conditions. Our simulation outputs show the importance of individualized services with appropriate timing and reveal a new method for personalizing psychotherapy to heterogeneous individuals. Future research is needed to expand the SD model to include additional mechanisms of depression.

## Supporting information

S1 Fig Sixteen categories of female participants. D_0_, R_0_, S_0_, and SI represent initial depressive symptoms and rumination, prior stressors, and ongoing stressors respectively.
(TIF)

S2 Fig Sixteen categories of male participants. D_0_, R_0_, S_0_, and SI represent initial depressive symptoms and rumination, prior stressors, and ongoing stressors respectively.
(TIF)

S1 Table Model formulations. (PDF)

## References

[1] World Health Organization. Depression and other common mental disorders: Global health estimates. Switzerland: WHO Document Production Services; 2017.

[2] Kessler RC, Berglund P, Demler O, Jin R, Koretz D, Merikangas KR, et al. National Comorbidity Survey Replication. The epidemiology of major depressive disorder: Results from the National Comorbidity Survey Replication (NCS-R). JAMA. 2003 Jun 18;289(23):3095–105. 12813115 10.1001/jama.289.23.3095

[3] Cuijpers P, Reynolds CF III, Donker T, Li J, Andersson G, Beekman A. Personalized treatment of adult depression: medication, psychotherapy, or both? A systematic review. Depress Anxiety. 2012 Oct;29(10):855–64. doi: 10.1002/da.21985 22815247

[4] Barber JP, Barrett MS, Gallop R, Rynn MA, Rickels K. Short-term dynamic psychotherapy versus pharmacotherapy for major depressive disorder: A randomized, placebo-controlled trial. J Clin Psychiatry. 2012 Jan;73(1):66–73. doi: 10.4088/JCP.11m06831 22152401

[5] Carter JD, McIntosh VV, Jordan J, Porter RJ, Frampton CM, Joyce PR. Psychotherapy for depression: A randomized clinical trial comparing schema therapy and cognitive behavior therapy. J Affect Disord. 2013 Nov;151(2):500–505. doi: 10.1016/j.jad.2013.06.034 23870427

[6] Simon GE, Ding V, Hubbard R, Fishman P, Ludman E, Morales L, et al. Early dropout from psychotherapy for depression with group- and network-model therapists. Adm Policy Ment Health. 2012 Nov;39(6):440–7. doi: 10.1007/s10488-011-0364-x 21710256 PMC3708590

[7] Barber JP, Muenz LR. The role of avoidance and obsessiveness in matching patients to cognitive and interpersonal psychotherapy: Empirical findings from the treatment for depression collaborative research program. J Consult Clin Psychol. 1996 Oct;64(5):951–8. doi: 10.1037//0022-006x.64.5.951 8916624

[8] DeRubeis RJ, Cohen ZD, Forand NR, Fournier JC, Gelfand LA, Lorenzo-Luaces L. The Personalized Advantage Index: translating research on prediction into individualized treatment recommendations. A demonstration. PLoS One. 2014 Jan 8;9(1):e83875. doi: 10.1371/journal.pone.0083875 24416178 PMC3885521

[9] Beutler LE, Engle D, Mohr D, Daldrup RJ, Bergan J, Meredith K, et al. Predictors of differential response to cognitive, experiential, and self-directed psychotherapeutic procedures. J Consult Clin Psychol. 1991 Apr;59(2):333–40. doi: 10.1037//0022-006x.59.2.333 2030196

[10] Cheavens JS, Strunk DR, Lazarus SA, Goldstein LA. The compensation and capitalization models: A test of two approaches to individualizing the treatment of depression. Behav Res Ther. 2012 Nov;50(11):699–706. doi: 10.1016/j.brat.2012.08.002 22982085

[11] Bousman CA, Forbes M, Jayaram M, Eyre H, Reynolds CF, Berk M, et al. Antidepressant prescribing in the precision medicine era: A prescriber’s primer on pharmacogenetic tools. BMC Psychiatry. 2017 Feb 8;17(1):60. doi: 10.1186/s12888-017-1230-5 28178974 PMC5299682

[12] Darabi N, Hosseinichimeh N. System dynamics modeling in health and medicine: A systematic literature review. Syst Dyn Rev. 2020 Jan;36(1):29–73.

[13] Homer JB, Hirsch GB. System dynamics modeling for public health: Background and opportunities. Am J Public Health. 2006 Mar;96(3):452–8. doi: 10.2105/AJPH.2005.062059 16449591 PMC1470525

[14] Sterman JD. Business dynamics: Systems thinking and modeling for a complex world. Boston, MA: Irwin/McGraw-Hill; 2000.

[15] Epstein JM. Why model?. J Artif Soc Soc Simul. 2008 Oct 31;11(4):12.

[16] Wittenborn AK, Rahmandad H, Rick J, Hosseinichimeh N. Depression as a systemic syndrome: Mapping the feedback loops of major depressive disorder. Psychol Med. 2016 Feb;46(3):551–62. doi: 10.1017/S0033291715002044 26621339 PMC4737091

[17] Richardson GP. Feedback thought in social science and systems theory. Waltham, MA: Pegasus Communications; 1999.

[18] Hosseinichimeh N, Wittenborn AK, Rick J, Jalali MS, Rahmandad H. Modeling and estimating the feedback mechanisms among depression, rumination, and stressors in adolescents. PLoS One. 2018 Sep 27;13(9): e0204389. doi: 10.1371/journal.pone.0204389 30261010 PMC6160072

[19] Wang PS, Berglund P, Olfson M, Pincus HA, Wells KB, Kessler RC. Failure and delay in initial treatment contact after first onset of mental disorders in the National Comorbidity Survey Replication. Arch Gen Psychiatry. 2005 Jun;62(6):603–13. doi: 10.1001/archpsyc.62.6.603 15939838

[20] McLaughlin KA, Nolen-Hoeksema S. Interpersonal stress generation as a mechanism linking rumination to internalizing symptoms in early adolescents. J Clin Child Adolesc Psychol. 2012;41(5):584–97. doi: 10.1080/15374416.2012.704840 22867280 PMC3465705

[21] Michl LC, McLaughlin KA, Shepherd K, Nolen-Hoeksema S. Rumination as a mechanism linking stressful life events to symptoms of depression and anxiety: longitudinal evidence in early adolescents and adults. J Abnorm Psychol. 2013 May;122(2):339–52. doi: 10.1037/a0031994 23713497 PMC4116082

[22] Coddington RD. The significance of life events as etiologic factors in the diseases of children: A survey of professional workers. J Psychosom Res. 1972 Jun;16(3):7–18. 5058990 10.1016/0022-3999(72)90018-9

[23] Cohen LH, Burt CE, Bjorck JP. Life stress and adjustment: Effects of life events experienced by young adolescents and their parents. Dev Psychol. 1987 Jul;23(4):583.

[24] Compas BE. Stress and life events during childhood and adolescence. Clin Psychol Rev. 1987 Jan 1;7(3):275–302.

[25] Abela JR, Brozina K, Haigh EP. An examination of the response styles theory of depression in third- and seventh-grade children: A short-term longitudinal study. J Abnorm Child Psychol. 2002 Oct;30(5):515–27. doi: 10.1023/a:1019873015594 12403154

[26] Kovacs M. Children’s Depression Inventory manual. NY: Multi-Health Systems; 1992.

[27] Ghaffarzadegan N, Epstein AJ, Martin EG. Practice variation, bias, and experiential learning in cesarean delivery: A data-based system dynamics approach. Health Serv Res. 2013 Apr;48(2 Pt 2):713–34. 23398502 10.1111/1475-6773.12040 PMC3626332

[28] Homer J, Milstein B, Wile K, Trogdon J, Huang P, Labarthe D, et al. Simulating and evaluating local interventions to improve cardiovascular health. Prev Chronic Dis. 2010 Jan;7(1): A18. 20040233 PMC2811513

[29] Lounsbury DW, Hirsch GB, Vega C, Schwartz CE. Understanding social forces involved in diabetes outcomes: A systems science approach to quality-of-life research. Qual Life Res. 2014 Apr;23(3):959–69. doi: 10.1007/s11136-013-0532-4 24062243 PMC8344372

[30] Milstein B, Homer J, Hirsch G. Analyzing national health reform strategies with a dynamic simulation model. Am J Public Health. 2010 May;100(5):811–9. doi: 10.2105/AJPH.2009.174490 20299653 PMC2853627

[31] Rahmandad H. Human growth and body weight dynamics: An integrative systems model. PLoS One. 2014 Dec 5;9(12):e114609. doi: 10.1371/journal.pone.0114609 25479101 PMC4257729

[32] Thompson KM, Tebbens RJ. Using system dynamics to develop policies that matter: Global management of poliomyelitis and beyond. Syst Dyn Rev. 2008 Dec;24(4):433–49.

[33] Tobias MI, Cavana RY, Bloomfield A. Application of a system dynamics model to inform investment in smoking cessation services in New Zealand. Am J Public Health. 2010 Jul;100(7):1274–81. doi: 10.2105/AJPH.2009.171165 20466963 PMC2882424

[34] Nolen-Hoeksema S, Stice E, Wade E, Bohon C. Reciprocal relations between rumination and bulimic, substance abuse, and depressive symptoms in female adolescents. J Abnorm Psychol. 2007 Feb;116(1):198–207. doi: 10.1037/0021-843X.116.1.198 17324030

[35] Ruscio AM, Gentes EL, Jones JD, Hallion LS, Coleman ES, Swendsen J. Rumination predicts heightened responding to stressful life events in major depressive disorder and generalized anxiety disorder. J Abnorm Psychol. 2015 Feb;124(1):17–26. doi: 10.1037/abn0000025 25688429 PMC4332541

[36] Nolen-Hoeksema S, Larson J, Grayson C. Explaining the gender difference in depressive symptoms. J Pers Soc Psychol. 1999 Nov;77(5):1061–72. doi: 10.1037//0022-3514.77.5.1061 10573880

[37] Hosseinichimeh N, Rahmandad H, Jalali MS, Wittenborn AK. Estimating the parameters of system dynamics models using indirect inference. Syst Dyn Rev. 2016 Apr;32(2):156–80.

[38] Keller MB, Coryell WH, Endicott J, Maser JD, Schettler PJ. Clinical guide to depression and bipolar disorder. Washington, D.C.: American Psychiatric Press; 2013.

[39] Timbremont B, Braet C, Dreessen L. Assessing depression in youth: relation between the Children’s Depression Inventory and a structured interview. J Clin Child Adolesc Psychol. 2004 Mar;33(1):149–57. doi: 10.1207/S15374424JCCP3301_14 15028549

[40] Clarke GN, Rohde P, Lewinsohn PM, Hops H, Seeley JR. Cognitive-behavioral treatment of adolescent depression: Efficacy of acute group treatment and booster sessions. J Am Acad Child Adolesc Psychiatry. 1999 Mar;38(3):272–9. doi: 10.1097/00004583-199903000-00014 10087688

[41] Ames CS, Richardson J, Payne S, Smith P, Leigh E. Mindfulness-based cognitive therapy for depression in adolescents. Child Adolesc Ment Health. 2014 Feb;19(1):74–78. doi: 10.1111/camh.12034 32878358

[42] Jellesma FC, Verkuil B, Brosschot JF. Postponing worrisome thoughts in children: The effects of a postponement intervention on perseverative thoughts, emotions and somatic complaints. Soc Sci Med. 2009 Jul;69(2):278–84. doi: 10.1016/j.socscimed.2009.04.031 19520470

[43] Carter JS, Garber J, Ciesla JA, Cole DA. Modeling relations between hassles and internalizing and externalizing symptoms in adolescents: A four-year prospective study. J Abnorm Psychol. 2006 Aug;115(3):428–42. doi: 10.1037/0021-843X.115.3.428 16866584

[44] Pettit JW, Lewinsohn PM, Seeley JR, Roberts RE, Yaroslavsky I. Developmental relations between depressive symptoms, minor hassles, and major events from adolescence through age 30 years. J Abnorm Psychol. 2010 Nov;119(4):811–24. doi: 10.1037/a0020980 21090879 PMC3058553

