# Supplementary figures and images for: Exploring personalized psychotherapy for depression: A system dynamics approach
Source: PLoS One. 2022 Oct 27;17(10):e0276441. doi: 10.1371/journal.pone.0276441 (PMC9612473; doi:10.1371/journal.pone.0276441)

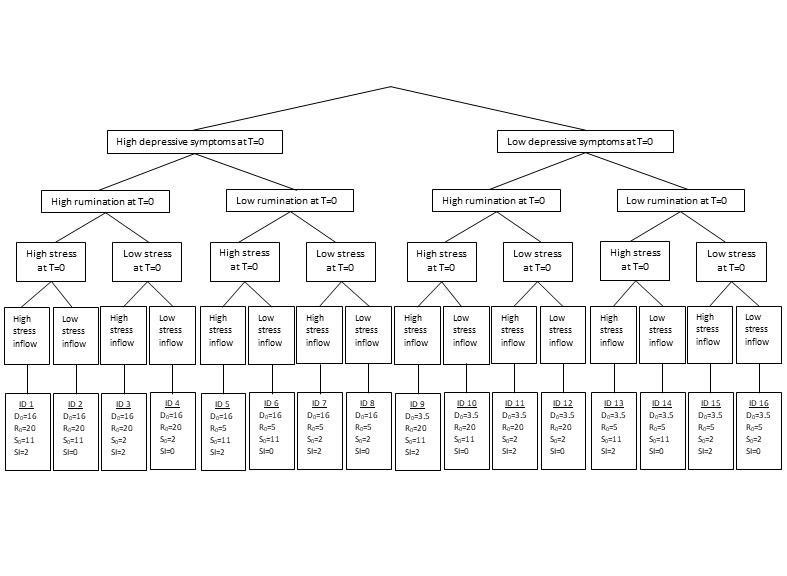

Supplement: S1 Fig — D0, R0, S0, and SI represent initial depressive symptoms and rumination, prior stressors, and ongoing stressors respectively. (TIF) [file pone.0276441.s001.TIF]

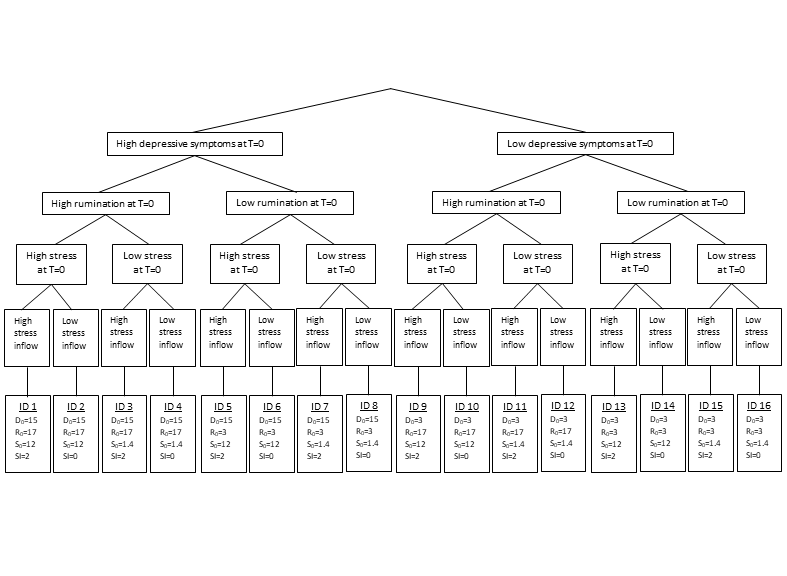

Supplement: S2 Fig — D0, R0, S0, and SI represent initial depressive symptoms and rumination, prior stressors, and ongoing stressors respectively. (TIF) [file pone.0276441.s002.TIF]
