# Supplementary material for: Exploring personalized psychotherapy for depression: A system dynamics approach
Source: PLoS One. 2022 Oct 27;17(10):e0276441. doi: 10.1371/journal.pone.0276441 (PMC9612473; doi:10.1371/journal.pone.0276441)
Supplement: S1 Table — (PDF) [file pone.0276441.s003.pdf]

S1 Table. Model Formulations.

**Formulations**	**Units**
*Adjusted Stressor0=Stressor0*Memory Time/Measurement time*	*engram*
Change in PinkNoise MDD=(WhiteNoise MDD-PinkNoise MDD) / Θ12	*DepScore/Month*
Change in PinkNoise Rum= (WhiteNoise Rum-PinkNoise Rum) / Θ12	*RumScore/Month*
Effect of Rumination on memory time=1.4741	*1/RumScore*
Fraction of Stimuli Negatively Perceived= 1	*Disruption/engram*
Gender=GET XLS CONSTANTS('AdolescentData.xlsx' , 'Sheet1' , 'B2')	*Dmnl*

*Indicated MDD=max(0, (Ɵ6+Ɵ7*Rumination) /(1-Ɵ8) + PinkNoise MDD*MDDSwitch)*	*DepScore*
*Indicated Rumination=max(0, (Ɵ1+Ɵ2*Depressive symptoms+Ɵ3*Gender+Ɵ4*Perceived Negative Stimuli)/(1-Ɵ5) +PinkNoise Rum*RumSwitch)*	*RumScore*
Let it go=(past stressors kept alive)/Memory Time	*engram/Month*
Depressive symptoms= SMOOTHI(Indicated MDD, Minimum Adjustment time/(1-Ɵ8) , MDD0)	*DepScore*
MDD0=GET XLS CONSTANTS('AdolescentData.xlsx' , 'Sheet1' , 'B4')	*DepScore*
MDDResidualMean=0	*DepScore*
MDDSwitch=0	*Dmnl*

Measurement time=6	*Month*
*Memory Time=Minimum Memory Time*Rumination* Ө9*	*Month*
Minimum Adjustment time=1	*Month*
Minimum Memory Time=1	*Month*
NoiseSeedMDD=3	*Dmnl*
NoiseSeedRum=2	*Dmnl*
*Perceived Negative Stimuli=past stressors kept alive *Fraction of Stimuli Negatively Perceived*	*Disruption*

; PinkNoise MDD(0)=0	*DepScore*
; PinkNoise Rum(0)=0	*RumScore*
Rum0= GET XLS CONSTANTS('AdolescentData.xlsx' , 'Sheet1' , 'B3')	*RumScore*
Rumination=SMOOTHI(Indicated Rumination, Minimum Adjustment time/(1-Θ5) , Rum0)	*RumScore*
RumResidualMean=0	*RumScore*
RumSwitch=0	*Dmnl*
Ongoing stressors=GET XLS CONSTANTS('AdolescentData.xlsx' , 'Sheet1' , 'B6')	*engram/Month*
; past stressors kept alive (0)=Adjusted Stressor0	*engram*

Stressor0=GET XLS CONSTANTS('AdolescentData.xlsx' , 'Sheet1' , 'B5')	engram
TIME STEP = 0.03125	Month
WhiteNoise MDD=RANDOM NORMAL(-10 , 10 , MDDResidualMean , 1 , NoiseSeedMDD)((ϴ11^2)*(2-(TIME STEP/ ϴ12)) /(TIME STEP/ ϴ12))^0.5*	*DepScore*
The formulation of white noise and pink noise are taken from Sterman (2000).	
WhiteNoise Rum=RANDOM NORMAL(-10 , 10 , RumResidualMean , 1 , NoiseSeedRum)((ϴ10^2)*(2-(TIME STEP/ ϴ12)) /(TIME STEP/ ϴ12))^0.5*	*RumScore*
ϴ1=-1.2504	*RumScore*
ϴ2=0.4236	*RumScore/DepScore*
ϴ3=2.5152	*RumScore*

Ө4=0.2518	*RumScore/Disruption*
Ө5=0.1639	*Dmnl*
Ө6=0.3730	*DepScore*
Ө7=0.0699	*DepScore/RumScore*
Ө8=0.8894	*Dmnl*
Ө9=1.4741	*1/ RumScore*
Ө10=7.8735	*Dmnl*
Ө11=0.0002	*Dmnl*

$\Theta 12=1.6008$	*Month*
